# Impacts of Stress Response and Negative Emotion on Mental Health of College Students During the COVID-19 Outbreak

**DOI:** 10.3389/fpsyt.2021.784661

**Published:** 2022-03-08

**Authors:** Fuming Xu, Long Huang

**Affiliations:** ^1^School of Education Science, Nanning Normal University, Nanning, China; ^2^School of Psychology, Jiangxi Normal University, Nanchang, China; ^3^School of Humanities and Management, Wannan Medical College, Wuhu, China

**Keywords:** COVID-19, epidemic stress response, negative emotion, mental health, epidemic panic

## Abstract

**Object:**

In this study, we aimed to explore the influences of stress responses and negative emotion on mental health of college students during the initial COVID-19 outbreak in China.

**Methods:**

A nationally representative sample of 1,351 college students, aged 18–23 years, took part in an online survey during the COVID-19 outbreak. The ANOVA, correlation coefficients, structural equation modeling (path analysis), and other statistical analysis methods were used for data analysis.

**Results:**

(1) The Chinese college students' epidemic panic and cognitive evaluation were found to be moderate (3.73 ± 0.74, 3.76 ± 0.62), while their defensive response was higher (4.61 ± 0.55). Their mental health during the COVID-19 outbreak was found to be good (3.75 ± 0.76). (2) The quality of their mental health was significantly and negatively associated with epidemic panic, and the quality of their mental health was significantly and positively associated with defensive response. (3) The quality of their mental health was significantly and negatively associated with negative emotion. (4) College students' epidemic panic and defensive response to the COVID-19 had a directly predictive effect on their mental health.

**Conclusion:**

College students' negative emotion played a partial mediating role in the relationship between epidemic panic and mental health. College students' negative emotion played a complete mediating role in the relationship between cognitive appraisal and mental health.

## Introduction

The COVID-19 pandemic was officially recognized as one of the greatest “public health emergencies in the world” by the WHO on January 31, 2020, and it reached pandemic status throughout the world on March 11, 2020. As of November 15, 2021, more than 254 million people had been infected worldwide, with a death toll exceeding 5.11 million according to the WHO. In addition, the COVID-19 pandemic has caused panic, anxiety, and depression among those affected by it. This series of chain reactions triggered by this negative emotion will further exacerbate the damage of the COVID-19 pandemic. The academic community has conducted empirical research on college students' stress, negative emotion, and mental health in previous major infectious disease outbreaks, for example, the SARS epidemic, which first broke out in China in 2003. Researchers explored the relationship between stress response, negative emotion, and mental health of the people during the SARS pandemic, which consistently found that there were significant correlations between people's stress, negative emotion, and mental health (1–3). The H1N1 pandemic, which first broke out in Mexico in 2009, quickly spread all over the world. Researchers explored the relationship between stress response, negative emotion, and mental health of the people during the H1N1 pandemic, which also found that there were significant correlations between people's stress, negative emotion, and mental health (4–8).

During the outbreak of COVID-19 in China, an investigation used the big data analysis method based on Sina Weibo and found that the public had increased negative emotion such as anxiety after January 20, 2020, in Wuhan, China. After the “city closure” on January 23, people were in a state of high stress response and negative emotion in the short term (9). A large-scale online survey on February 13–16, 2020, in China, which used self-rating anxiety scale (SAS) and self-rating depression scale (SDS), found that people's anxiety and depression were slightly higher than the norm (but not clinically significant), and there was no significant difference between people in Hubei province and other regions (10). This result confirmed the findings of Tong (11) on anxiety and depression among college students in the SARS epidemic, who found that the SAS and the depression scale (CESD) did not distinguish college students' difference of emotional responses between the epidemic core region and other regions. However, Wen et al. conducted a survey, which used the SAS from January 24 to February 8, 2020, and found that the anxiety level of the people in the core region was significantly higher than that of the people in other regions. On the one hand, this difference may be difficult to accurately measure the mental health of the affected people using a single self-rating scale of anxiety and depression. On the other hand, the mental health problems may be more directly related to their stress response and negative emotion. Most of the above relevant studies only measured the level of anxiety or depression (12).

**Figure 1:**
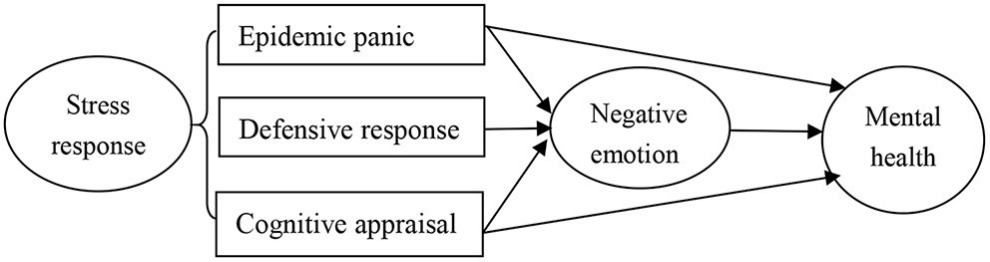
Relationship model between stress response, negative emotion, and mental health.

According to the stress coping theory (13, 14), the external event of “city closure” or mandatory quarantine could be seen as a stressor that initiates stress response (e.g., defensive behavior and cognitive appraisal). Such appraisal might result in negative emotions (e.g., worry about the COVID-19) and thereby endanger mental health. Therefore, this study attempts to construct a relationship model between college students' stress response to the COVID-19, negative emotion, and mental health (Figure 1). When facing the outbreak of COVID-19, college students will have different kinds and degrees of stress responses, as shown in Figure 1. On the one hand, college students' stress response may have a direct impact on their mental health. On the other hand, college students' stress response may have an indirect impact on mental health through the mediating role of negative emotion. The primary objective of this study was to investigate the associations between stress response, negative emotion, and mental health status among college students during the initial phase of the COVID-19 outbreak in China.

## Methods

### Participants and Design

An anonymous cross-sectional survey was conducted from February 1 to 10 (the 8–17 days after Chinese New Year and during winter vacation for college students), 2020, by using online questionnaires. A snowball sampling strategy was adopted, and 1,351 valid samples were collected. There were 306 college students in Hubei Province, 413 college students in Anhui, Henan, and Jiangxi around Hubei Province, and 632 college students in other provinces far away from the epidemic, such as Guangxi, Guangdong, Heilongjiang, Zhejiang, and Beijing. There are 927 female college students and 424 male college students. The average age of the subjects was 21.75 ± 9.81 years. The inclusion criterion was that the subjects needed to be full-time college students. The exclusion criteria included the following: (a) self-reported COVID-19 diagnosis (*n* = 5) and (b) failure to pass the internal consistency checks (*n* = 23). It was specified on the questionnaire that the return of the completed questionnaire implied that informed consent had been given. Ethical approval was obtained from the ethics committee of the corresponding author's affiliated university.

### Measures

#### General Health Questionnaire

The General Health Questionnaire (GHQ-12) was composed of 12 items, which had good reliability and validity (15–17). The Likert 5-point scoring was used. The data of 675 participants were used to conduct a confirmatory factor analysis. The chi-square value = 47.59, DF = 31, *p* = 0.06, chi-square value/DF = 1.54, GFI = 0.99, AGFI = 0.97, NFI = 0.99, IFI = 1.00, CFI = 1.00, RMSEA = 0.03. The Cronbach's α coefficient of the GHQ-12 was 0.86.

#### Stress Response Questionnaire

The questionnaire was developed by Tong (11) and had a total of 13 items, which were divided into three dimensions: cognitive appraisal, epidemic panic, and defensive response to the epidemic. The SARS in each item was replaced by COVID-19 in this study. Confirmatory factor analysis of the stress response questionnaire showed that the chi-square value = 58.67, DF = 38, *p* = 0.06, chi-square value/DF = 1.54, GFI = 0.99, AGFI = 0.97, NFI = 0.97, IFI = 0.99, CFI = 0.99, and RMSEA = 0.03. The Cronbach's α coefficient of cognitive appraisal subscale was 0.79; the Cronbach's α coefficient of epidemic panic subscale was 0.82; the Cronbach's α coefficient of defensive response subscale was 0.83; and the Cronbach's α coefficient of the entire questionnaire was 0.81.

#### Negative Emotion Self-Assessment Questionnaire

Referring to the PANAS scale (18, 19), six specific emotions were used to investigate the emotional feelings of college students when facing the COVID-19 epidemic. The six negative emotions are “tension, worry, panic, anger, sadness, and anxiety.” We used Likert-style five-point scoring. Firstly, the data of 676 subjects were used to conduct exploratory factor analysis. One factor with a feature root >1 was extracted, and the total interpretation rate was 70.49%. Then, confirmation factor analysis was performed on the data of the remaining 675 subjects and found that the chi-square value = 55.15, DF = 17, *p* = 0.06, chi-square value/DF = 2.76, GFI = 0.98, AGFI = 0.96, NFI = 0.97, IFI = 0.98, CFI = 0.99, and RMSEA = 0.05. The Cronbach's α coefficient is 0.91.

### Statistical Analysis

The ANOVA was used to test the significance of between-group differences. Pearson correlations were used to test the associations between mental health and its related influencing factors. A structural equation model (path analysis) with full information likelihood estimation was used to test the hypothesized mediation model for mental health. Tests for the direct, indirect, and total effects were based on 2,000 bootstrapped samples. Effect estimates and bias-corrected 95% confidence intervals (CI) were derived. The indices of good fit included the root mean square error of approximation (RMSEA) <0.06 and comparative fit index (CFI) >0.95. Analyses were conducted using SPSS 22.0 and AMOS 22.0. A two-sided *p* below 0.05 was considered statistically significant.

## Results

### Stress Response and Mental Health of College Students Facing COVID-19

Firstly, the results of stress responses and mental health of college students when facing the outbreak of COVID-19 were as shown in Table 1. The level of defensive response of college students was found to be higher. The levels of both epidemic panic and cognitive appraisal were found to be moderate. The level of mental health of college students was found to be good.

**Table 1:** Stress response and mental health of college students when facing COVID-19.

	**Epidemic**	**Defensive**	**Cognitive**	**Mental**
	**panic**	**response**	**appraisal**	**health**
*M* ±*SD*	3.73 ± 0.74	4.61 ± 0.55	3.76 ± 0.62	3.75 ± 0.76

Secondly, an ANOVA was conducted on the stress response and mental health of college students in different regions. The results showed that there were significant regional differences in college students' epidemic panic [*F*_(2, 1, 348)_ = 27.70, *p* < 0.001, ${\text{η}}_{\text{p}}^{2}$ = 0.04]. College students in Hubei province felt higher levels of epidemic panic (*M* = 3.97, *SD* = 0.75) than did college students in non-adjacent provinces (*M* = 3.75, *SD* = 0.71) (*p* < 0.001). College students in non-adjacent provinces felt higher levels of epidemic panic than did college students in neighboring provinces (*M* = 3.55, *SD* = 0.73) (*p* < 0.001). There were significant regional differences in college students' defensive response [*F*_(2, 1, 348)_ = 3.41, *p* < 0.05, ${\text{η}}_{\text{p}}^{2}$ = 0.01]. College students in Hubei province had more defensive responses (*M* = 4.70, *SD* = 0.58) than college students in both non-adjacent provinces (*M* = 4.60, *SD* = 0.52) and adjacent provinces (*M* = 4.58, *SD* = 0.57) (*p* < 0.05). There were significant regional differences in college students' cognitive appraisal [*F*_(2, 1, 348)_ = 22.57, *p* < 0.001, ${\text{η}}_{\text{p}}^{2}$ = 0.03]. College students in Hubei province had higher cognitive appraisal (*M* = 3.98, *SD* = 0.62) than college students in non-adjacent provinces (*M* = 3.75, *SD* = 0.61) (*p* < 0.001). College students in non-adjacent provinces had higher cognitive appraisal than college students in adjacent provinces (*M* = 3.65, *SD* = 0.59) (*p* < 0.05). However, the results showed that there was no significant regional difference in college students' mental health (*p* > 0.05).

### Relationship Between Stress Response, Negative Emotion, and Mental Health

Firstly, we tested the correlations between college students' stress responses, negative emotion, and mental health (Table 2). The results showed that there was a significant negative correlation between college students' mental health and epidemic panic. There was a significant positive correlation between college students' mental health and defensive response. There was a significant negative correlation between college students' mental health and negative emotion.

**Table 2:** Correlation between stress response, negative emotion, and mental health.

	**1**	**2**	**3**	**4**	**5**
1. Epidemic panic	1				
2. Defensive response	0.22^***^	1			
3. Cognitive appraisal	0.67^***^	0.31^***^	1		
4. Negative emotion	0.52^***^	0.13^***^	0.47^***^	1	
5. Mental health	−0.25^***^	0.09^***^	−0.05	−0.44^***^	1

*** *p < 0.001*.

**Figure 2:**
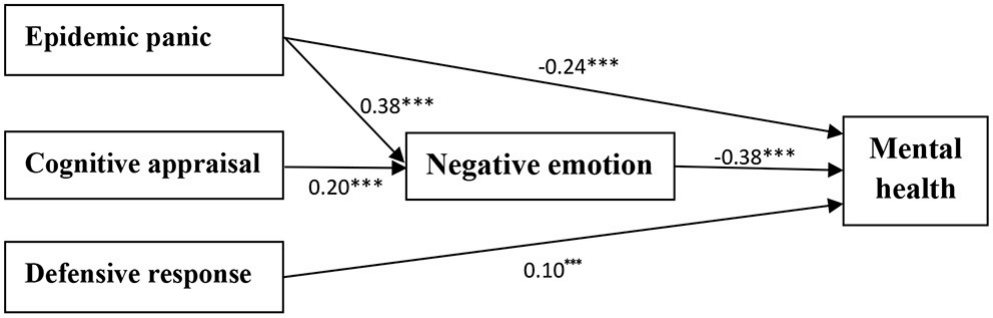
Relationship between epidemic stress response, negative emotion, and mental health. ***p < 0.001.

Secondly, we used a structural equation model (path analysis) to construct the relationship model between epidemic stress response, negative emotion, and mental health of college students (Figure 2). The chi-square value = 3.43, DF = 3, *p* = 0.33, chi-square value/DF = 1.14, GFI = 1.00, AGFI = 0.99, NFI = 1.00, IFI = 1.00, CFI = 1.00, RMSEA = 0.01. On the one hand, college students' epidemic panic and defensive response had a directly predictive effect on the mental health. On the other hand, negative emotion not only played a partial mediating role in the relationship between epidemic panic and mental health but also played a complete mediating role in the relationship between cognitive appraisal and mental health.

## Discussion

Generally speaking, we found that Chinese college students' stress response and mental health were relatively mild when facing the outbreak of COVID-19. This might be the case because both Chinese spring festival and winter vacation played a double-buffering role during the COVID-19 epidemic in China. The further testing of college students' stress response and mental health from different regions found that, compared with college students in other provinces, college students in Hubei province who were in the epidemic core area had bigger stress responses and worse mental health. The results were consistent with the results of Wen et al. (20), which found that the perceived risk and anxiety level of people in Hubei province were significantly higher than those of other regions. In addition, this might be the case because of the control characteristics of the COVID-19 epidemic in China. The regional characteristics of the COVID-19 outbreak were very obvious, which belong to a single-core area, namely, Hubei province and Wuhan city. Therefore, most college students' stress response is obviously lower than those in Hubei province and Wuhan city. This difference was also in line with the “ripple effect.” The ripple effect means that the closer to the core area of the crisis the people are, the higher their risk perception and negative emotion, and the greater the impact they feel (21). However, the result was not fully consistent with the ripple effect. We found that college students far away from Hubei province and Wuhan city had bigger stress responses to COVID-19 than those in neighboring provinces. This might be the case because the effectiveness and control of epidemic prevention were very good. Except for Hubei province, the severity of the COVID-19 epidemic in other provinces was similar and safe.

In addition, we found that there was a significant correlation between college students' stress responses, negative emotion, and mental health. That is to say, college students' stress responses had a significantly predictive effect on negative emotion and mental health. The result was consistent with previous studies, which also found that the stress response to SARS and the worry about SARS significantly predicted the level of anxiety and depression of college students (3, 11). When facing COVID-19, college students' epidemic panic and defensive response had a directly predictive effect on the level of mental health, in which the predictive effect of epidemic panic is negative, while the predictive effect of defensive response is positive. It was easy to understand that college students' epidemic panic was not conducive to the maintenance of their mental health, while college students' self-protective defense response was helpful to the maintenance of their mental health.

The results further revealed that college students' negative emotion played a mediating role in the relationship between stress response and mental health. The result was consistent with the prior research, which showed that, during the initial COVID-19 outbreak in China, Chinese college students' emotional distress played a partial mediating role in the relationship between infection risk and mental health (22). This result was partially consistent with a related study, which found that fear of COVID-19 played a mediating role in the relationship between family cohesion and stress consequences (23). In particular, college students' negative emotion played a partial mediating role in the relationship between epidemic panic and mental health. College students' negative emotion played a complete mediating role in the relationship between cognitive appraisal and mental health. This showed that the different relationship between different dimensions of stress response, negative emotion, and mental health.

## Limitations And Future Research

Our survey belongs to the domain of quantitative research and lacks qualitative analysis. In addition, our results draw on cross-sectional data using a structural equation model; although we recruited a large sample, this design cannot be used to draw conclusions about causal relationships. Future research will require the use of a longitudinal survey.

## Conclusion

College students' epidemic panic and defensive response to COVID-19 had a directly predictive effect on their mental health. College students' negative emotion played a partial mediating role in the relationship between epidemic panic and mental health. College students' negative emotion played a complete mediating role in the relationship between cognitive appraisal and mental health.

## Data Availability Statement

The original contributions presented in the study are included in the article/supplementary material, further inquiries can be directed to the corresponding author/s.

## Ethics Statement

The studies involving human participants were reviewed and approved by Academic Ethics Committee, School of Educational Sciences, Nanning Normal University. The patients/participants provided their written informed consent to participate in this study.

## Author Contributions

FX conceived and designed the review. LH and FX wrote the manuscript. Both authors contributed to the article and approved the submitted version.

## Funding

This study was supported by the National Natural Science Foundation of China (Grants Nos. 72164028 and 71971103), and MOE (Ministry of Education in China) Project of Humanities and Social Sciences (Grants No. 20YJC190006).

## Conflict of Interest

The authors declare that the research was conducted in the absence of any commercial or financial relationships that could be construed as a potential conflict of interest.

## Publisher's Note

All claims expressed in this article are solely those of the authors and do not necessarily represent those of their affiliated organizations, or those of the publisher, the editors and the reviewers. Any product that may be evaluated in this article, or claim that may be made by its manufacturer, is not guaranteed or endorsed by the publisher.

## References

[1] Qian M Ye D Dong W Zhang L Liu X Zhang X. Behavior, cognition and emotion of the public in Beijing towards SARS. J Chin Mental Health. (2003) 17:515–20. (Chinese). 10.3321/j.issn:1000-6729.2003.08.001

[2] Shi K Fan H Jia J Li W Song Z Gao J. Chinese people's perception of SARS information and psychological behavior. Acta Psycho Sinica. (2003) 35:546–54. (Chinese). 10.1023/A:102228950970232214707

[3] Xie X Zheng R Xie D Wang H. SARS psychological panic phenomenon analysis. J Peking Univers Natural Sci Ed. (2005) 4:628–38. (Chinese). 10.3321/j.issn:0479-8023.2005.04.01733503049

[4] Goodwin R Haque S Neto F Myers LB. Initial psychological responses to Influenza A, H1N1 (“Swine flu”). BMC Infect Dis. (2009) 9:166. 10.1186/1471-2334-9-166 19807908 PMC2765446

[5] Jones JH Salathe M. Early assessment of anxiety and behavioral response to novel swine-origin influenza A(H1N1). PLoS ONE. (2009) 4:e8032. 10.1371/journal.pone.0008032 19997505 PMC2779851

[6] Rubin GJ Amlot R Page L Wessely S. Public perceptions, anxiety, and behavior change in relation to the swine flu outbreak: Cross sectional telephone survey. BMJ. (2009) 339:b2651. 10.1136/bmj.b2651 19574308 PMC2714687

[7] Liao Q Cowling BJ Lam WW Ng DM Fielding R. Anxiety, worry and cognitive risk estimate in relation to protective behaviors during the 2009 influenza A/H1N1 pandemic in Hong Kong: ten cross-sectional surveys. BMC Infect Dis. (2014) 14:169. 10.1186/1471-2334-14-169 24674239 PMC3986671

[8] Lau JT Griffiths S Choi KC Tsui HY. Avoidance behaviors and negative psychological responses in the general population in the initial stage of the H1N1 pandemic in Hong Kong. BMC Infect Dis. (2010) 10:139. 10.1186/1471-2334-10-139 20509887 PMC2891756

[9] Li S Wang Y Xue J Zhao N Zhu S. The impact of COVID-19 epidemic declaration on psychological consequences: a study on active weibo users. Int J Environ Res Public Health. (2020) 17:2032. 10.3390/ijerph17062032 32204411 PMC7143846

[10] Liu D Ren Y Li Y Yu X Qu W Wang Z. Psychological status of Chinese residents during COVID-19 outbreak: an online cross-sectional study. Chin J Psychiatry. (2021) 3:181–9. (Chinese). 10.3760/cma.j.cn113661-20200302-0008234054603

[11] Tong H. “SARS” stress response model and its characteristics. Acta Psycho Sinica. (2004) 36:103–9. (Chinese).

[12] Wang X Wang X Ma H. Evaluation manual of mental health scale. Chinese J Mental Health. (1999). (Chinese).

[13] Folkman S Lazarus RS. Stress, Appraisal, and Coping. New York, NY: Springer (1984).

[14] Lazarus RS. Evolution of a model of stress, coping, and discrete emotions. In Rice VH, editor. Handbook of Stress, Coping, and Health: Implications for Nursing Research, Theory, and Practice. Thousand Oaks, CA: Sage (2000). p. 195–222.

[15] Li Y Li Y. Multi sample analysis of the structure of 12 item general health questionnaire (GHQ-12). Psychol Explor. (2015) 4:355–9. (Chinese). 10.3969/j.issn.1003-5184.2015.04.012

[16] Tahara M Mashizume Y Takahashi K. Coping mechanisms: Exploring strategies utilized by Japanese healthcare workers to reduce stress and improve mental health during the COVID-19 pandemic. Int J Environ Res Public Health. (2021) 18:131. 10.3390/ijerph18010131 33375444 PMC7795636

[17] Liu M Dufour G Sun Z Galante J Xing C Zhan J. The impact of the COVID-19 pandemic on the mental health of young people: a comparison between China and the United Kingdom. Chin J Traumatol. (2021) 24:231–6. 10.1016/j.cjtee.2021.05.005 34074594 PMC8343781

[18] Watson D Clark LA Tellegen A. Development and validation of brief measures of positive and negative affect: The PANAS scales. J Pers Soc Psychol. (1988) 54:1063–70. 10.1037/0022-3514.54.6.1063 3397865

[19] Zhang W Diao J Schick CJ. The cross-cultural measurement of positive and negative affect. Psychol Sci. (2004) 27:77–9. (Chinese). 10.3969/j.issn.1671-6981.2004.01.02024230921

[20] Wen F Ma S Ye H Qi Y Zuo B. “Psychological typhoon eye effect” and “ripple effect:” double perspective test of risk perception and anxiety characteristics of people in different COVID-19 severity regions. Acta Psycho Sinica. (2020) 52:1087–104. (Chinese). 10.3724/SP.J.1041.2020.01087

[21] Slovic P. Perception of risk. Science. (1987) 236:280–5. 10.1126/science.3563507 3563507

[22] Zeng Y Ye B Zhang Y Yang Q. Family cohesion and stress consequences among Chinese college students during COVID-19 pandemic: a moderated mediation model. Front Public Health. (2021) 9:703899. 10.3389/fpubh.2021.703899 34336777 PMC8319383

[23] Xin M Luo S She R Yu Y Li L Wang S. Negative cognitive and psychological correlates of mandatory quarantine during the initial COVID-19 outbreak in China. Am Psychol. (2020) 75:607–17. 10.1037/amp0000692 32673008

